# Practical Use of Bacteria

**Published:** 1892-09

**Authors:** 


### Practical Use of Bacteria.

It is reported that a few weeks ago a plague of mice threatened to destroy the whole harvest of Thessaly, in Greece. The Greek Government asked Professor Loffler, of Greiswald, to assist it, which he effectually did by inoculating some of the mice and turning them loose, thus causing a fatal epidemic among them.

